# Design and Fabrication of Micro Hemispheric Shell Resonator with Annular Electrodes

**DOI:** 10.3390/s16121991

**Published:** 2016-11-25

**Authors:** Renxin Wang, Bing Bai, Hengzhen Feng, Ziming Ren, Huiliang Cao, Chenyang Xue, Binzhen Zhang, Jun Liu

**Affiliations:** 1 Science and Technology on Electronic Test & Measurement Laboratory, North University of China, Taiyuan 030051, China; wangrenxin@nuc.edu.cn (R.W.); bai402728119@163.com (B.B.); nuc_fenghengzhen@163.com (H.F.); 18234157705@163.com (Z.R.); caohuiliang@nuc.edu.cn (H.C.); xuechenyang@nuc.edu.cn (C.X.); 2 Key Laboratory of Instrumentation Science & Dynamic Measurement, Ministry of Education, North University of China, Taiyuan 030051, China

**Keywords:** hemispheric shell resonator, annular electrodes, glassblowing, equivalent gap distance

## Abstract

Electrostatic driving and capacitive detection is widely used in micro hemispheric shell resonators (HSR). The capacitor gap distance is a dominant factor for the initial capacitance, and affects the driving voltage and sensitivity. In order to decrease the equivalent gap distance, a micro HSR with annular electrodes fabricated by a glassblowing method was developed. Central and annular cavities are defined, and then the inside gas drives glass softening and deformation at 770 °C. While the same force is applied, the deformation of the hemispherical shell is about 200 times that of the annular electrodes, illustrating that the deformation of the electrodes will not affect the measurement accuracy. S-shaped patterns on the annular electrodes and internal-gear-like patterns on the hemispherical shell can improve metal malleability and avoid metal cracking during glass expansion. An arched annular electrode and a hemispheric shell are demonstrated. Compared with HSR with a spherical electrode, the applied voltage could be reduced by 29%, and the capacitance could be increased by 39%, according to theoretical and numerical calculation. The surface roughness of glass after glassblowing was favorable (*R*q = 0.296 nm, *R*a = 0.217 nm). In brief, micro HSR with an annular electrode was fabricated, and its superiority was preliminarily confirmed.

## 1. Introduction

A Coriolis Vibratory Gyroscope (CVG) is a sensor that can measure the angle and the angular velocity by the change in vibration mode due to Coriolis Effect from the resonator’s rotation [1]. It is desirable because it has a variety of advantages compared to the classic gyroscope, including smaller size, lighter weight, lower power dissipation, shorter starting time, and larger time-constant.

With the continuous development of society and technology, miniature products are demanded for people’s livelihood and military applications [2]. Micro Electro Mechanical Systems (MEMS) can achieve the miniaturization of CVGs, and Micro machined Vibratory Gyroscopes (MVGs) currently appear widely on the market because the fabrication of MEMS can make them low cost, small size, and with batch production. Nowadays, the majority of MVGs are on the rate-grade level of performance; the others are on the level of tactical-grade performance. However, it is difficult to obtain MVGs that can reach inertial-grade performance [2]. In addition, although MVGs have been studied and fabricated using silicon as structural material and features defined by photolithography and deep reactive ion etching (DRIE) techniques, the complicated processes and the whole performances of MVGs have recently been intractable problems.

The macro-size Hemispherical Resonator Gyroscope (HRG) is used in inertial systems, and the most successful HRG was developed by Northrop Grumman [3,4]. The structure of the HRG belongs to axisymmetric shell resonator gyroscopes with contactless scheme. It is often used on spacecraft such as airplanes and even space shuttles to monitor and control their flying posture. Some HRGs that include a specific performance index (for example, overloading-resistibility) can be used to guide missiles to hit a target precisely. The basic principle of the HRG can be summed up as: when the hemispheric shell rotates in a condition of resonance, the vibration mode moves relatively on the shell because of the Coriolis Effect, and the relative angular velocity on the shell has a proportional relation with the carrier angular velocity.

In recent years, the combinations of the HRG and MEMS have been researched, and micro-size HRGs (μHRG) has attracted great attention. The first challenge in the fabrication of μHRGs is how to fabricate a micro-size hemispheric shell resonator (HSR), which is composed of a hemispheric shell and surrounding electrodes. Using conventional techniques to fabricate a micro-size wineglass or umbrella shell is impossible. Therefore, approaches for the fabrication of a 3D-MEMS resonator were researched by the use of glassblowing, blow molding (to form an umbrella shell or a birdbath shell) [5,6,7], thin films of polysilicon [8,9], SiO_2_ [10], diamond [11], and some other techniques. A novel micro-scale fused silica (FS) shell gyroscope called the birdbath resonator gyroscope (BRG) has been introduced [7]. Polysilicon HSRs are demonstrated [8,9], the difference being that the electrodes are fabricated by P-doped silicon [8] and polysilicon [9], respectively. A novel structure was designed and fabricated by glassblowing [12,13]. The shell is M-shaped, and the shell material is fused silica. Compared with Pyrex glass, fused silica glass has higher Q-factor, which is important for the inertial component. However, fused silica also has a higher melting point (1700 °C), so it is difficult to use glassblowing to form an M-shaped shell. The next challenge is how to drive the HSR and detect its deformation. A method of driving and detecting the HSR to use a capacitance effect was discussed in [14,15]. Out-of-plane electrostatic transduction on MEMS wineglass resonators was demonstrated in [16]. The last challenge is how to integrate the hemispheric shell and electrodes with precise position. Using a glassblowing technique, electrode hemispheres were also fabricated around the hemispheric shell, and metal was sputtered on the surface of the electrodes and the hemispheric shell to form capacitors [17].

**Figure 1:**
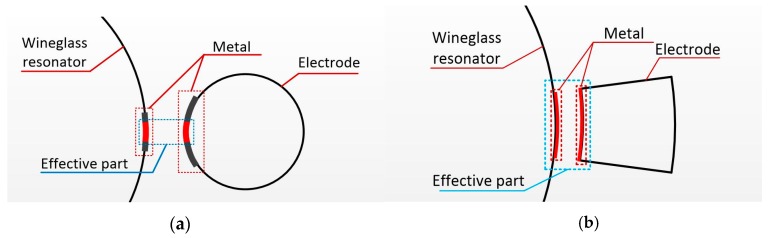
(**a**) The shape of electrode in [6]; (**b**) The shape of electrode in this paper.

The whole performance of HSR mostly depends on the material and the parameters of the hemispheric shell. Several methods for the fabrication of MEMS wineglass resonators have been published in the literature. These methods include: thermally growing oxide in isotropically etched cavities [8], micro crystalline diamond deposition on hemispherical molds [18], and utilizing a sphere mold on which polysilicon and ultra low expansion titania silicate glass are deposited and releasing the hemispherical shell [19]. However, these methods involve complicated processes, and the roughness of hemispheric shells is difficult to reduce down to 10 nm due to fabrication precision of the molds. Alternately, a glassblowing technique was introduced to form Pyrex 7740 [6] glass, titania silicate glass [20], or a fused silica shell [16] which had the advantages of an ultra-smooth surface owing to its thermoplastic process. In addition, the shape of the surrounding electrode should be considered, because it has an influence on the performance of HSR. Glassblowing an HSR with tiny hemispheric electrodes was presented in [21], bringing about a large equivalent gap distance, as shown in Figure 1a.

**Figure 2:**
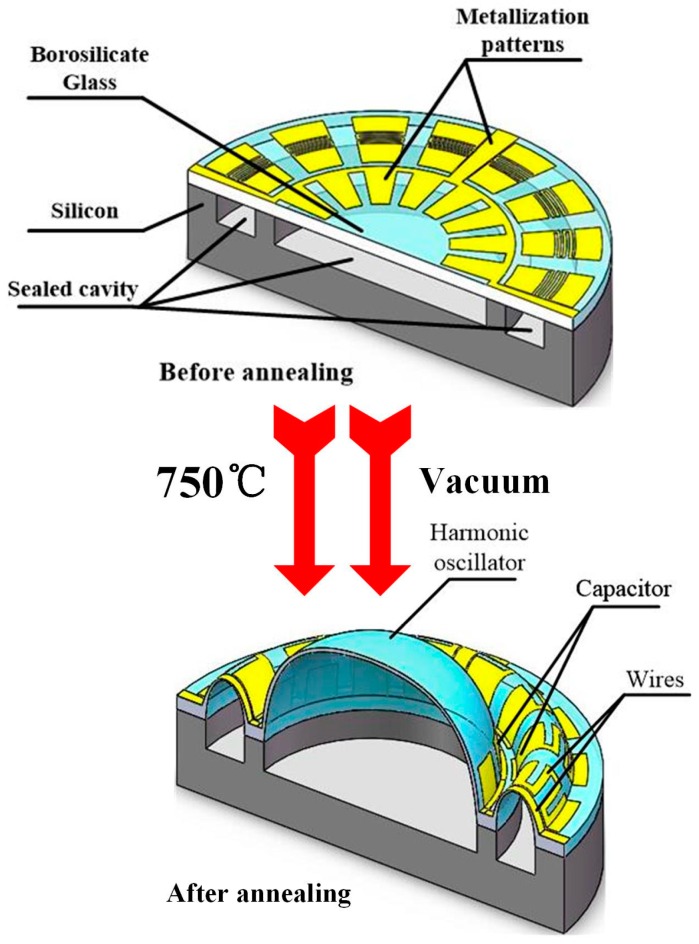
Schematic diagram of glassblowing process and metallization patterns used to form capacitors.

Therefore, this paper explores a structure that has annular electrodes around the hemispheric shell (equivalent gap distance is smaller than that in Figure 1a, as shown in Figure 1b) for the fabrication of glassblowing and blow molding (Figure 2), and this fabrication can realize self-aligned hemispheric shell and electrodes. On the glass layer, the metallization patterns are demonstrated as S-shaped to improve metal malleability during annealing. The effective area between the hemispheric shell and the annular electrode is constant, and the gap of the counter-electrode is minimized. So, this structure guarantees that the drive signal act on the hemispheric shell easily, and the detect signal can be collected accurately.

## 2. Design and Simulation

### 2.1. The Relationship between Driving Force and Electrode Gap

To simplify the case, a parallel plate capacitor is taken as an example.

The potential energy *U*:

(1) $$U=-\frac{1}{2}CV^{2}$$

where *C* is the capacity value, and *V* is the voltage applied on electrodes.

(2) $$C=\frac{\varepsilon A}{g-x}$$

*ε* is the permittivity of capacitor, *A* is the parallel area, *g* is the initial gap, and *x* is the relative displacement of the electrode.

(3) $$F_{e}=-\frac{\partial U}{\partial x}=\frac{\partial C}{\partial x}\frac{V^{2}}{2}=\frac{\varepsilon A}{2{\left(g-x\right)}^{2}}V^{2}$$

In the case of x<<g, it could be induced that:

(4) $$F_{e}=\frac{\varepsilon A}{2g^{2}}V^{2}$$

Especially, for a curved capacitor,

(5) $$F_{e}=\int \frac{\varepsilon }{2g^{2}}V^{2}dA=\frac{\varepsilon A}{2g_{\mathrm{eq}}^{2}}V^{2}$$

where *g*_eq_ is the equivalent gap distance. This indicates that F is in direct proportion to 1/*g*_eq_ when *A* and *V* are constant.

Correspondingly, the capacitance is in reverse proportion to the equivalent gap distance, which determines the sensitivity of detection.

### 2.2. The Parameters Simulation of the Hemispherical Shell

**Figure 3:**
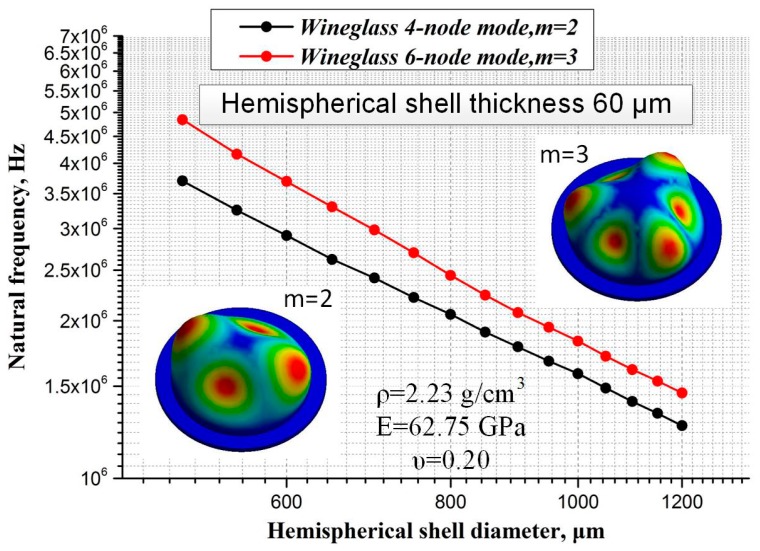
The relationship of natural frequencies and hemispherical shell diameter for mode *n* = 2, 3.

The mode shapes and resonant frequencies of this hemispherical structure are analyzed by modal analysis in finite element analysis (FEA) software (ANSYS Workbench). The material of the hemispherical structure is Pyrex 7740 (Corning Inc., Corning, NY, USA), and the parameters of Pyrex 7740 are defined as follows: density *ρ* = 2.23 g/cm^3^, Young’s modulus *E* = 62.75 GPa, and Poisson’s Ratio υ = 0.20. The thickness of the hemispherical shell is defined to be about 60 μm, and the shell diameter ranges from 500 μm to 1200 μm, with its corresponding height ranging from 250 μm to 650 μm. Figure 3 shows the correlation between hemispherical shell diameters and natural frequencies in the vibration mode m = 2, 3. Actually, the main purpose of the simulation is to figure out the structure parameters, which is important for process and layout design. Besides, the results indicate that a strictly inverse relationship exists between natural frequency and hemispherical shell diameter.

### 2.3. The Interaction of Hemispherical Shell and Annular Electrodes

**Figure 4:**
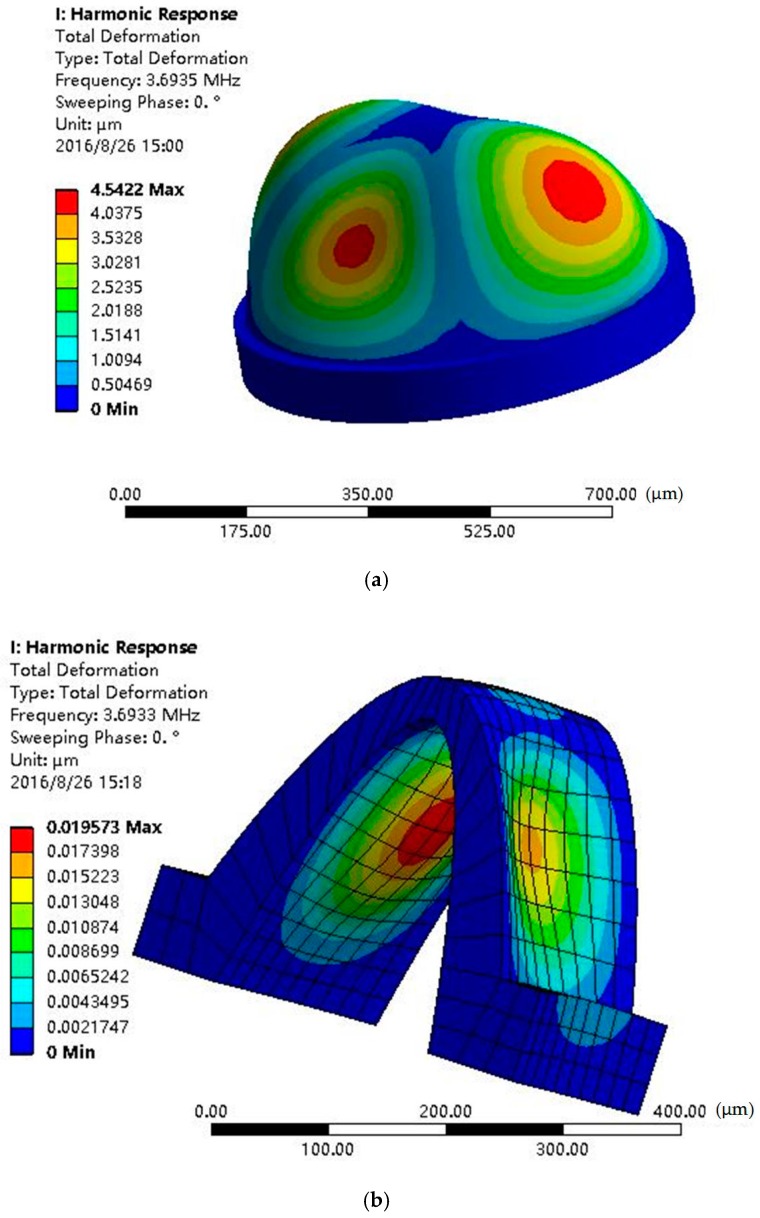
Applied with the same force, max deformations of the shell (**a**) and electrode (**b**) are 4.542 μm and 0.019 μm, respectively.

At the working state, the vibration of the shell is caused by capacitive excitation, and the gyro signal is detected by the capacitance, so the distance between the shell and electrodes is the key factor. Because the action of forces is mutual, when the shell is vibrated, the electrode’s deformation occurs simultaneously. Harmonic response analysis is used to simulate the total deformation of the shell-exerted force for the natural frequency. Figure 4 shows the deformation of shell and electrodes applied with the same force, and Figure 4a indicates that the max total deformation of the shell is 4.542 μm at the 3.693 MHz resonant frequency. Figure 4b shows that the electrode max deformation is 0.019 μm at the same frequency. Compared with the shell deformation, the electrode deformation is small enough to be negligible.

## 3. Fabrication

**Figure 5:**
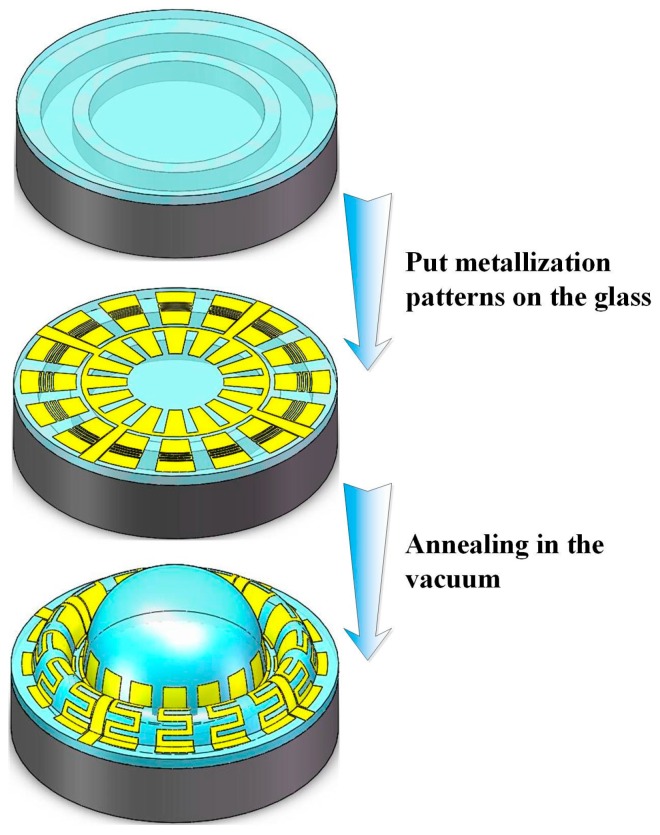
General fabrication of annular electrodes and the hemispheric shell.

**Figure 6:**
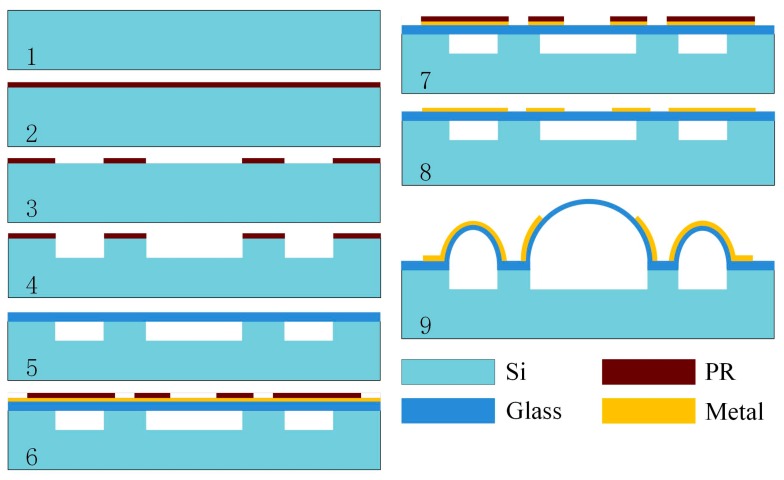
Detailed fabrication of making annular electrodes and the hemispheric shell. 1: Prepare the wafer; 2: Spin the photoresist; 3: Photolithography; 4: DRIE; 5: Clean wafer and bond glass wafer; 6: Sputter metal and photolithography; 7: Etch metal; 8: Clean the wafer; 9: Anneal the wafer to form annular electrodes and hemispheric shell.

The forming process of the annular electrodes is similar to that of the hemispheric shell, utilizing the pressure difference between the inside and outside of the hermetic cavity and the surface tension forces from the softened glass. The fabrication process for annular electrodes and the hemispheric shell structure is shown in Figure 5 and Figure 6, and involves: (1) Cavity formation via inductively coupled plasma (ICP) etching technology; (2) anodic bonding of the glass layer to the silicon wafer with cavities, and thinning and polishing the glass layer; (3) sputtering metal on top of the glass layer and ion beam etching (IBE) to form metallic patterns; (4) annealing and forming annular electrodes and the hemispheric shell.

The first step of the fabrication process is photolithography. Before this process, the wafer is cleaned by a standardized cleaning process to eliminate organics, and is preprocessed by Hexamethyl Disilazane (HMDS) to increase the adhesion of the silicon wafer and photoresist. An approximately 7 μm-thick AZ P4620 photoresist (AZ Electronic Materials USA Corp., Branchburg, NJ, USA) layer is spun on the silicon wafer, and then the mask is formed. Cylindrical cavities and annular cavities are etched in the silicon wafer using deep reactive ion etching (DRIE) about 100 μm deep.

The next step is anodic bonding. First, the remaining photoresist on the silicon wafer after etching is removed with acetone or with the use of a plasma stripper. Then, a borosilicate glass wafer is cleaned by a standardized cleaning process and bonded to the silicon wafer under normal atmospheric pressure via anodic bonding machine, forming a sealed cavity structure. Next, the glass is thinned and polished to about 100 μm thickness.

After that, metallization patterns are defined on the glass layer. The metallization pattern must be ductile enough to avoid out-of-step plastic deformation during the glassblowing step. Therefore, at the beginning, Cr, Cu, and Au are sputtered on the glass layer with about 100 nm, 600 nm, and 400 nm layer thicknesses, successively. Then, 2 μm-thick AZ P5214 photoresist (AZ Electronic Materials USA Corp.) is spun and patterned on the Au layer. The three metals are etched by IBE, and remaining photoresist on the Au layer is removed.

The last step of the process is annealing in vacuum and glassblowing. The temperature is raised up to soften point of borosilicate glass (about 750–770 °C) for 120–180 s in vacuum and retained for 150–300 s. Next, the wafer is cooled down rapidly to room temperature for 300 s, and the annular electrodes and hemispheric shell are finally fabricated.

## 4. Results and Discussions

**Figure 7:**
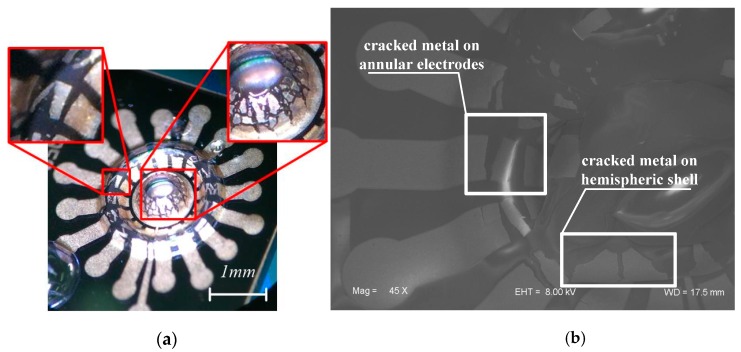
Photographs of initially fabricated annular electrodes and hemispheric shell. Not only the metal on center shell is cracked, but also the annular electrode. (**a**) is observed by optical microscope; and (**b**) is by Scanning Electron Microscope (SEM).

**Figure 8:**
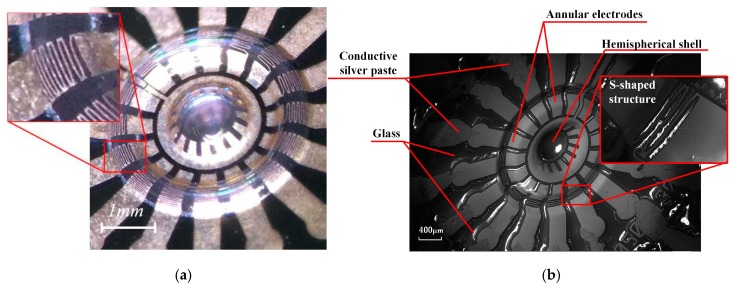
Photographs of optimized annular electrodes and hemispheric shell. Metal traces are not broken. (**a**) is observed by optical microscope; and (**b**) is by SEM.

At first, the metal on the annular electrode is straight without an S-shaped pattern. The glassblowing process makes glass extend and arch to form the shell and annular structure. Correspondingly, the metal pattern on the structure is also dramatically stretched at the same time. The maximum deformation of the metal line can reach up to π/2, which may cause the metal line to crack, as shown in Figure 7. In addition, the continuously patterned metal on the hemisphere is also severely cracked. To solve this problem, the S-shape pattern is optimized on the annular electrode and an internal-gear-like pattern is proposed on the hemispherical shell, which could dramatically release the extension stress and keep the metal in continuous condition (Figure 8).

In order to demonstrate the inner structure, HSR is split along the silicon cleavage facet. The shell was split manually rather than by machine. Hence, the nonuniformity of the margin is due to manual splitting rather than a fault in glassblowing.

**Figure 9:**
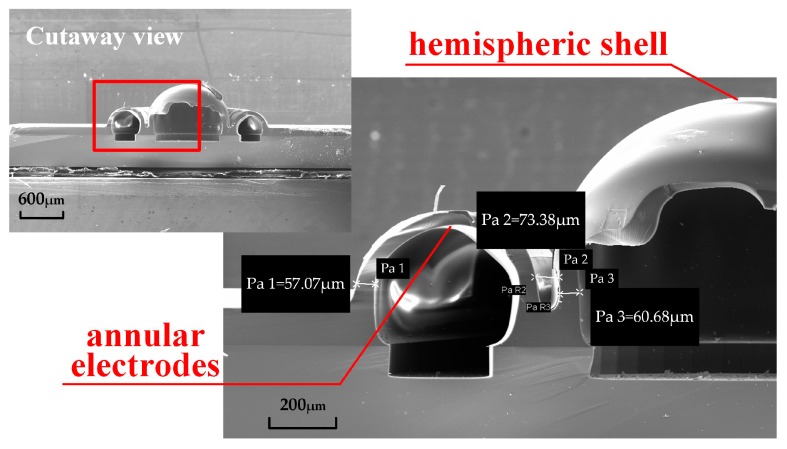
Cutaway view of annular electrodes and hemispheric shell.

The cutaway view of HSR is measured as shown in Figure 9. An arched annular electrode and a hemispheric shell are obviously observed, consistent with the design in Figure 2. Because cleavage fracture would not occur on brittle amorphous glass, an irregular fracture surface of the hemispheric shell is in accordance with expectation. Furthermore, the thickness of the annular electrodes and the hemispherical shell are measured to be 57.07 μm and 60.68 μm, respectively. The electrode gap is about 73.38 μm. As a matter of fact, the parameters used in the simulation are kept in accordance.

The electrostatic force *F* in [17] and for our device were calculated. We built a model with the shell diameter *R* = 1000 μm, the effective area *A* = 400 μm × 260 μm, the diameter of spherical electrode *r_e_* = 500 μm, the minimum distance between the shell and electrodes *D* = 70 μm. After numerical calculation, *F*_s_ = 3.5 × 10^−11^·*V*^2^ in [17]. In this paper, *F*_a_ = 6.8 × 10^−11^·*V*^2^. So, the annular electrode structure is favorable for the improvement of *F*. In the simulation, when the electrostatic pressure is 1 Pa, the deformation of the shell is about 4.5 μm, reaching up to 6% of the original gap. Thus, the capacitance change due to deformation could be remarkably measured. To produce the force *F* = 1 Pa × 400 μm × 260 μm = 1.04 × 10^−7^ N, the applied voltage *V* can be calculated. The *V*_s_ (voltage applied on the spherical electrode) is figured out as 55 V and *V*_a_ (voltage applied on the annular electrode) = 39 V, meaning that the applied voltage could be decreased by 29%.

Correspondingly, the capacitance could be also calculated numerically, showing that it could be increased by 39%.

**Figure 10:**
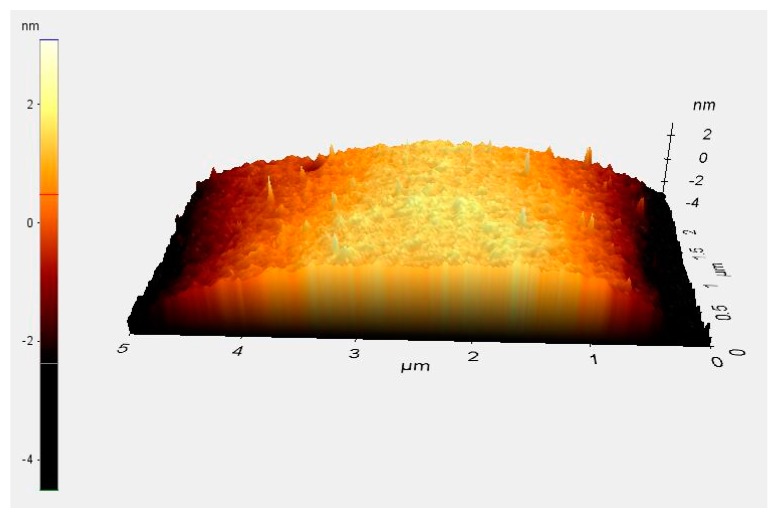
Atomic force microscopy (AFM) graph of glass surface after annealing.

Surface roughness can affect hemispherical shell vibration and Q-factor [6,22]. After annealing, the surface roughness was characterized by atomic force microscope (AFM), as shown in Figure 10. The results demonstrate that the hemispherical shell surface root-mean-square (rms) roughness (*R*q) was 0.296 nm, and the average roughness (*R*a) was 0.217 nm. As a comparison, the rms surface roughness was 0.85 nm in [6] and 0.51 nm in [22], respectively. It should be noted that the roughness values show progress compared to that of various molding-based approaches (rarely below 10 nm).

## 5. Conclusions

We have fabricated a micro hemispherical shell resonator with annular electrodes, integrating electrostatic driving and capacitive detection transducers. The annular electrodes make it possible to decrease the driving voltage and increase the sensitivity by decreasing the capacitor equivalent gap distance, compared to hemispheric electrodes. The shell deformation was 4.542 μm at the 3.693 MHz resonant frequency, and the electrode max deformation was 0.019 μm at the same frequency. In contrast with the shell deformation, the electrode deformation was negligible. The hemispherical shell and annular electrodes were fabricated by glassblowing, and the integration of the hemispherical shell and electrodes was implemented by self-aligned technology. An S-shaped pattern on the annular electrode and an internal-gear-like pattern on the shell were designed in order to avoid metal cracking. Theoretical and numerical calculation indicated advancements in the reduction of applied voltage and the promotion of capacitance. It is illustrated that the annular electrode is actuated and the shell was hemispheric as designed. The surface roughness was characterized as *R*q = 0.296 nm, *R*a = 0.217 nm, which is much less than that of molding-based approaches. These results indicate this HSR has prospective application for μHRGs. It should be noted that this study focuses on the design and fabrication of HSR, and further research on the elaborate characterization of the resonator is necessary.
